# Food restriction promotes damage reduction in rat models of type 2 diabetes mellitus

**DOI:** 10.1371/journal.pone.0199479

**Published:** 2018-06-20

**Authors:** Carlos Vinicius Dalto da Rosa, Jéssica Men de Campos, Anacharis Babeto de Sá Nakanishi, Jurandir Fernando Comar, Isabela Peixoto Martins, Paulo Cézar de Freitas Mathias, Maria Montserrat Diaz Pedrosa, Vilma Aparecida Ferreira de Godoi, Maria Raquel Marçal Natali

**Affiliations:** 1 Department of Morphological Sciences, State University of Maringá, Paraná, Brazil; 2 Department of Biochemistry, State University of Maringá, Maringá, Paraná, Brazil; 3 Department of Biotechnology, Cell Biology and Genetics State University of Maringá, Paraná, Brazil; 4 Department of Physiological Sciences, State University of Maringá, Maringá, Paraná, Brazil; Stellenbosch University, SOUTH AFRICA

## Abstract

There are several animal models of type 2 diabetes mellitus induction but the comparison between models is scarce. Food restriction generates benefits, such as reducing oxidative stress, but there are few studies on its effects on diabetes. The objective of this study is to evaluate the differences in physiological and biochemical parameters between diabetes models and their responses to food restriction. For this, 30 male Wistar rats were distributed in 3 groups (n = 10/group): control (C); diabetes with streptozotocin and cafeteria-style diet (DE); and diabetes with streptozotocin and nicotinamide (DN), all treated for two months (pre-food restriction period). Then, the 3 groups were subdivided into 6, generating the groups CC (control), CCR (control+food restriction), DEC (diabetic+standard diet), DER (diabetic+food restriction), DNC (diabetic+standard diet) and DNR (diabetic+food restriction), treated for an additional two months (food restriction period). The food restriction (FR) used was 50% of the average daily dietary intake of group C. Throughout the treatment, physiological and biochemical parameters were evaluated. At the end of the treatment, serum biochemical parameters, oxidative stress and insulin were evaluated. Both diabetic models produced hyperglycemia, polyphagia, polydipsia, insulin resistance, high fructosamine, hepatic damage and reduced insulin, although only DE presented human diabetes-like alterations, such as dyslipidemia and neuropathy symptoms. Both DEC and DNC diabetic groups presented higher levels of protein carbonyl groups associated to lower antioxidant capacity in the plasma. FR promoted improvement of glycemia in DNR, lipid profile in DER, and insulin resistance and hepatic damage in both diabetes models. FR also reduced the protein carbonyl groups of both DER and DNR diabetic groups, but the antioxidant capacity was improved only in the plasma of DER group. It is concluded that FR is beneficial for diabetes but should be used in conjunction with other therapies.

## Introduction

Diabetes mellitus is a chronic disease that has become an epidemic. It is estimated that more than 420 million adults are affected worldwide with this disease, and this number increases alarmingly [1]. This situation stems from problems related to the modern lifestyle, which include high intake of processed foods, bigger elderly population, reduced physical activity and obesity [2].

The most frequent type of diabetes is type 2 (T2DM) or independent insulin, characterized mainly by chronic hyperglycemia and insulin resistance in peripheral tissues [3]. In T2DM pancreatic beta cells produce insufficient amounts of insulin to maintain normoglycemia [4] or produce excessive amounts due to failure in the peripheral tissues insulin response, which generates insulin resistance [3]. Among the complications of T2DM, oxidative stress has great relevance. Hyperglycemia and metabolic dysregulation increase the production of reactive oxygen species (ROS), damaging tissues [5].

Despite the various forms of study of T2DM in animal models [6–10], few studies aim to compare parameters and treatments between different animal models [11]. In addition to T2DM models based on genetic alterations [11], models involving chemical substances [12], diet alterations [13] or both [14] have been used. Differences between models, mainly related to metabolic changes, can be critical in choosing the best model for the study of a particular treatment.

Changes in diet alone hardly lead to T2DM in rats [15,16]. Therefore, the development of a model of T2DM with chronic characteristics demands the alliance of streptozotocin and altered diet [7,17]. On the other hand, some models that use variations of the chemical substances for diabetes induction seem to generate less characteristics similar to the human condition of T2DM [18–20] when compared to the models involving dietary alterations [10].

Food restriction (FR) consists of reducing food intakewhile preserving minimum levels of nutrients. FR has already shown benefits for pancreatic beta cell function, maintenance of blood glucose and other factors in patients with T2DM [21,22] and in animal models [9,23]. Therefore, FR could be a less invasive alternative in the control of T2DM compared to other interventions such as bariatric surgeries, which have become more frequent due to the epidemic of obesity and T2DM [24,25].

The oxidative stress has been associated with the development and progression of diabetes mellitus and its complications. This was demonstrated by increases in the production of reactive oxygen species (ROS) associated to a diminished capacity of the antioxidant system in many tissues of both patients and experimental diabetic animals [26–28]. The blood interacts with all tissues of the body and then the oxidative status of the plasma reflects at least in part the oxidative status of the whole body. Therefore, the oxidative stress can be evaluated in the plasma of diabetic rats with the purpose of presenting an overview of the whole body oxidative status in models of T2DM. Hence, this study aimed to evaluate the effects of FR on two distinct T2DM models, induced by streptozotocin and cafeteria style diet or with streptozotocin and nicotinamide, through general physiological characterization, blood biochemical analysis and insulin production evaluations.

## Materials and methods

### Drugs and chemicals

Streptozotocin, nicotinamide and anti-insulin antibodies (AB 260137) used in this study were obtained from Sigma-Aldrich, USA. The Optium Xceed glucometer and dosing strips were purchased from Abbott, Brazil. Thionembutal was supplied by the Abbott laboratory, USA. The blood laboratory test kits were supplied by Gold Analisa Diagnostics Ltda., Brazil. Recombinant human insulin was obtained from PerkinElmer, Shelton, CT, USA. All reagents used had the best possible quality.

### Animals and treatment

Thirty male Wistar rats (*Rattus novergicus*, 90 days, 328.2±21.8 g of initial body mass), from the Central animal house of the State University of Maringá, were kept individually in polypropylene boxes, with light and dark cycles of 12 hours and temperature of 22±2°C in the Sectorial animal house of the Department of Morphological Sciences. All procedures related to the animals followed the standards established by the Ethics Commission on the Use of Animals (protocol number 7590050415/2015), in order to minimize the suffering of animals.

After one week of acclimatization, the animals were treated for a total duration of 4 months, divided into 2 periods: months 1 and 2 (**pre-food restriction**) and months 3 and 4 (**food restriction**).

Initially, during the pre-food restriction period the animals were divided into 3 groups (n = 10/group): C (control), DE (type 2 diabetes + diet) and DN (type 2 diabetes + nicotinamide). Group C rats received only intravenous saline, and were fed with standard diet and water *ad libitum*.

The diabetization of the DE group rats consisted of intravenous injection of streptozotocin (STZ—35mg/kg) dissolved in citrate buffer (10mM, pH 4.5) after overnight fasting. After confirming hyperglycemia, the animals received a cafeteria-style diet (33% standard ration Nuvilab^®^, 33% Nestlé^®^ condensed milk and 7% sugar and water), sugar water (32%) and normal water, *ad libitum* (adapted from Sahin et al.[15] and Trammel et al.[29]).

The diabetization of the DN group rats consisted of the initial intravenous injection of STZ (60 mg/kg), and after fifteen minutes intraperitoneal injection of nicotinamide (NIC-80 mg/kg). After seven days they received a new dose of STZ (30 mg/kg), and after fifteen minutes, 40 mg/kg of NIC (adapted from Sharma et al.[19]). After confirming hyperglycemia, these animals received standard diet and water *ad libitum*.

Both diabetic models used produce moderate insulin insufficiency [30]. The confirmation of the diabetic state occurred one week after these protocols, checking the fasting glycemia. Animals with stable glycemia greater than 200 mg/dL of blood were considered diabetic (T2DM) [7].

In the food restriction period, group C was subdivided into groups CC (control) and CCR (control + food restriction with standard diet); the DE group, in DEC (diabetic + standard diet) and DER (diabetic + food restriction with standard diet); and DN formed DNC (diabetic + standard diet) and DNR (diabetic + dietary restriction with standard diet) (n = 5/group) (Table 1).

**Table 1:** Diets of experimental groups during 4 months of treatment.

Months 1 and 2 (pre food restriction period)		Months 3 and 4 (food restriction period)	
**Group C**	Standard diet (*ad libitum)*	**Group CC**	Standard diet (*ad libitum)*
		**Group CCR**	Standard diet (16 g)
**Group DE**	Cafeteria-style diet + sugar water (32%) (*ad libitum)*	**Group DEC**	Standard diet (*ad libitum)*
		**Group DER**	Standard diet (16 g)
**Group DN**	Standard diet (*ad libitum)*	**Group DNC**	Standard diet (*ad libitum)*
		**Group DNR**	Standard diet (16 g)

After the subdivision, groups CC, DEC and DNC received standard diet and water *ad libitum* in the period of months 3 and 4. The CCR, DER and DNR groups were submitted to a food restriction protocol (FR), which consisted of receiving only 50% of the average food intake of the control group (C), which served as the basis for all groups. Therefore, animals under FR received 16 g of standard diet daily, and water *ad libitum* (Table 1). Throughout the treatment it was monitored: daily consumption of food; weekly body mass; and biweekly water consumption and fasting/postprandial blood glucose were measured. During the entire period, the animals were weekly monitored for adverse clinical signs, like hypoglycemia and excessive weight loss, based on these parameters.

### Assessments of pre- and food restriction periods and tissue collection

At the end of the pre-food restriction (pre-FR) period (2 first months of treatment), glucose tolerance (GTT) and insulin tolerance (ITT) tests were performed. Three days after these tests, the animals were anesthetized with intravenous ketamine/xylazine (100/10 mg.Kg^-1^) injection, and 1 mL of blood was collected by cardiac puncture from each rat. The collected blood was used for the measurement of insulin by radioimmunoassay and analysis by means of specific kits of the following biochemical parameters: fructosamine, total proteins, total cholesterol, triglycerides, AST (aspartate aminotransferase), ALT (alanine aminotransferase) and alkaline phosphatase.

At the end of the FR period (4 months of treatment), GTT and ITT were again obtained. Then, the animals were intraperitoneally anesthetized (40 mg/kg of body mass) with intraperitoneal thionembutal and had 5 mL of blood collected by cardiac puncture. All animals died from hypovolemic shock. The blood collected during these experiments was centrifuged for 10 minutes at 3000 rpm to obtain the supernatant. The serum collected was stored in a freezer at -80 °C until use.

For the final serological analysis, insulin was evaluated by radioimmunoassay and the following biochemical parameters with a fraction of serum collected: fructosamine, total proteins, albumin, total cholesterol, HDL and VLDL cholesterols, triglycerides, AST, ALT and alkaline phosphatase. Another portion of the serum was analyzed for oxidative stress.

After the euthanasia of the animals, we also collected abdominal fats for weighing: retroperitoneal, mesenteric, periepididymal and subcutaneous. The pancreas was also collected and fixed in 4% paraformaldehyde for 6 hours. After the fixation, the material was embedded in paraffin for the preparation of histological slides with cuts destined to the immunohistochemical technique for the evaluation of the insulin-producing pancreatic cells.

#### Glucose tolerance test (GTT) and Insulin tolerance test (ITT)

For evaluation of the GTT glycemic curve, a solution of glucose (1.5 g.Kg^-1^) was administered to rats, at night fasting, via gavage. Then, the glycemia was measured with a glucometer at 0, 5, 10, 15, 30, 45 and 60 minutes.

The ITT curve was obtained after application of an intraperitoneal injection of regular insulin (Novolin^®^; 1 U kg^-1^, Novo Nordisk, Montes Claros, Brazil) to rats at 2-hour fasting. The glycemia was then measured with a glucometer at 0, 5, 10, 15, 20, 25, 30 and 60 minutes.

For both techniques, was obtained a constant of increase of the glycemic rate (for GTT), and of decay of the glycemic rate (for ITT), kGTT and kITT respectively [31].

#### Plasma oxidative status

The total antioxidant capacity (TAC) of the plasma was measured by spectrophotometry using 2,2’-azino-bis(3-ethylbenzthiazoline-6-sulphonic acid) or ABTS [32]. TAC was calculated from the standard curve prepared with Trolox, a water-soluble analog of vitamin E, and the results were expressed as nmol.(mL plasma)^-1^.

Plasmatic thiol contents were measured by spectrophotometry (412 nm) using DTNB (5,5’-dithiobis 2-nitrobenzoic acid) as previously described [32]. Thiol contents were calculated using the molar extinction coefficient (ε) of 1.36 × 10^4^•M^−1^.cm^−1^ and expressed as nmol.(mg protein)^−1^. Protein carbonyl groups were measured by spectrophotometry using 2,4-dinitrophenylhydrazine [33]. The levels of protein carbonyl groups were calculated using the molar extinction coefficient (ε) of 2.20 × 10^4^ M^−1^.cm^−1^ and expressed as nmol.(mg protein)^−1^. The groups of carbonylated proteins were measured by spectrophotometry using 2,4-dinitrophenylhydrazine. The calculation was done using the molar extinction coefficient (ε) of 2.20 x 10^4^ M^-1^.cm^-1^ and expressed as nmol.(mg of protein)^-1^.

#### Analysis of insulin-producing pancreatic cells

For this immunohistochemical analysis, pancreas samples were submitted to standard histological treatment, with dehydration in increasing concentrations of alcohol, diaphanization in xylol and inclusion in paraffin. The included tissue was cut into a microtome (Leica^®^ RM2245) to obtain semiserial 5 μm thick sections. The immunostaining process, aiming at the labeling of insulin, included stages of hydration, endogenous peroxidase blockade, primary antibody reaction, Meyer hematoxylin counter-staining and, finally, Permount slide mounting. The images were captured under light microscope under 40x objective (Olympus BX41, Olympus America Inc., New York, USA) coupled to high resolution camera (Olympus Q Color 3 Olympus America Inc., New York, USA). Image Pro Plus, version 4.5 (Media Cybernetics, Silver Spring, MD) was used for the analysis of the images and the percentage of immunoreactive cells for insulin was analyzed in 30 areas of 50x50 μm of pancreatic islets per animal, in which the positive and negative insulin cells were counted, resulting in an insulin-positive cell marking index.

#### Dosage of blood insulin

Plasma insulin concentrations were determined by radioimmunoassay (RIA) [34] using the Wizard2, TM-2470 automatic gamma counter (PerkinElmer, Shelton, CT, USA). RIA was made using a human insulin standard, a rat anti-insulin antibody, and a radiolabeled recombinant human insulin (125). The coefficients of intra and interassay variation varied by 12.2 and 9.8%, respectively. The limit of detection was 1033 pmol/L.

### Statistical analysis

The data were initially submitted to the Kolmogorov-Smirnov test to verify normality. Once the data were normal, the data were submitted to One-way Variance Analysis (ANOVA) followed by Tukey’s *post-hoc* test. The number of immunolabelled cells for pancreatic islet insulin generated non-parametric data that were analyzed under the Kruskal-Wallis test and Dunns *post-hoc* test. The results were presented as mean±standard error (SE) of the mean and level of significance of 5% (p<0.05). Data were statistically evaluated using GraphPad Prism (GraphPad Software, version 5.1, San Diego, CA, USA).

## Results

### Physiological parameters

**Fig 1:**
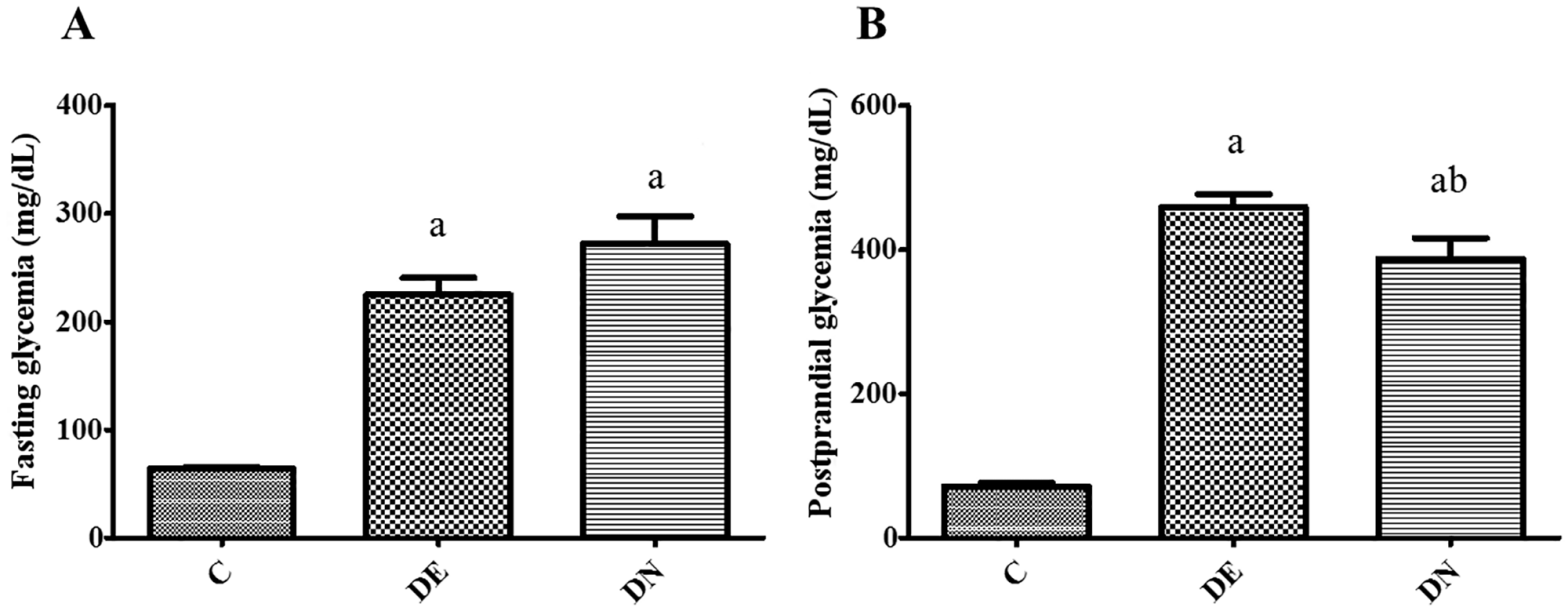
Pre-food restriction glycemia. The glycemia of the rats was evaluated in the fasting (A) and postprandial (B) states after the initial 2 months of treatment for the control groups (C), diabetic+streptozotocin+cafeteria-style diet (DE) and diabetic+streptozotocin+nicotinamide (DN). Results expressed as mean±SE (n = 7-10/group). a p<0.05 vs C; b p<0.05 vs DE. One-way ANOVA and Tukey’s *post-hoc* test.

**Fig 2:**
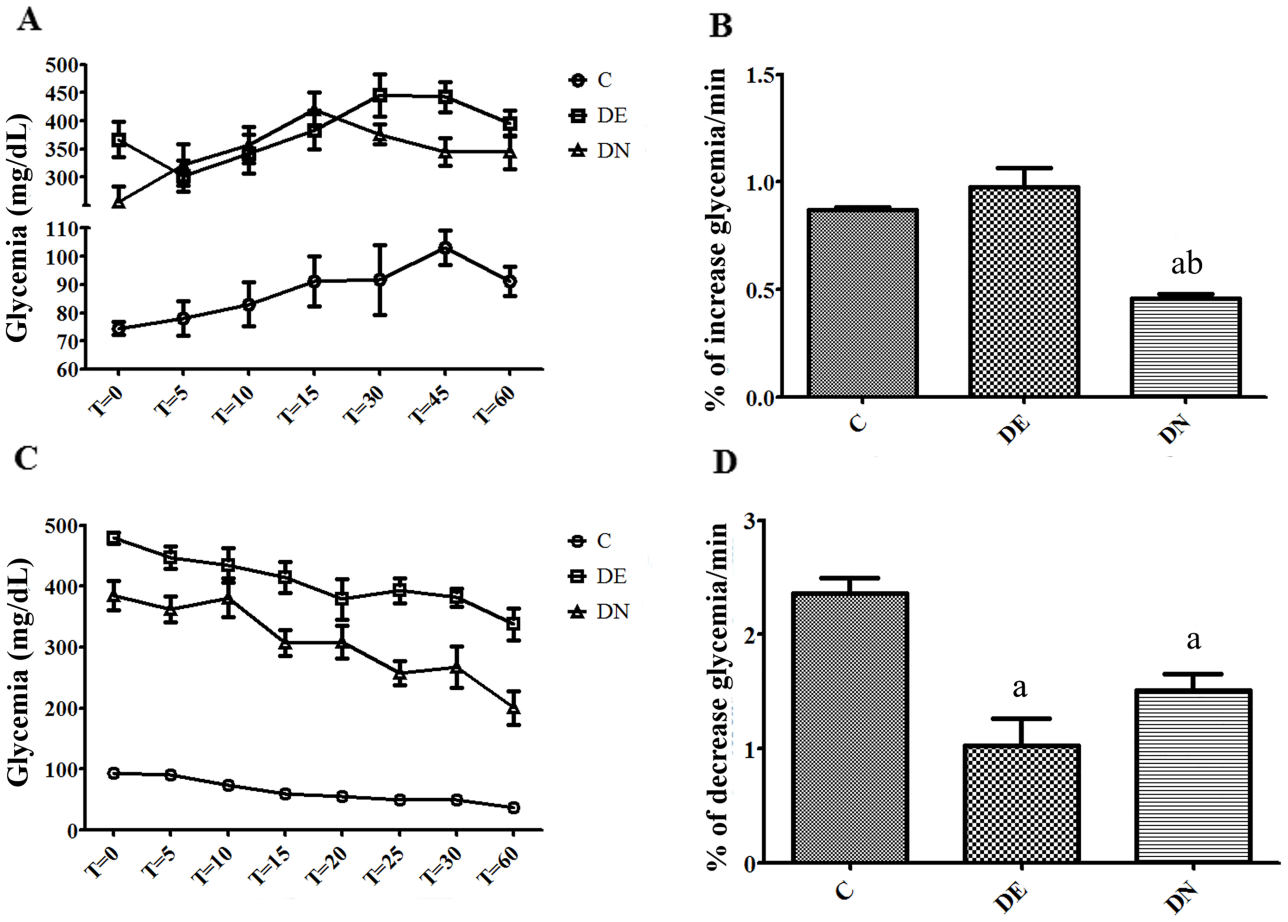
Glucose tolerance and insulin resistance pre-food restriction of rats (after 2 months of initial treatment). (A) Glucose tolerance test (GTT); (B) Rate of increase of glycemia per minute during GTT; (C) insulin-tolerance test (ITT); (D) Rate of blood glucose reduction per minute during ITT. Groups: control (C), diabetic+streptozotocin+cafeteria style diet (DE) and diabetic+streptozotocin+nicotinamide (DN). Results expressed as mean±SE (n = 5/group). a p<0.05 *vs* group C; b p<0.05 *vs* DE; one-way ANOVA and Tukey’s *post-hoc* test.

**Fig 3:**
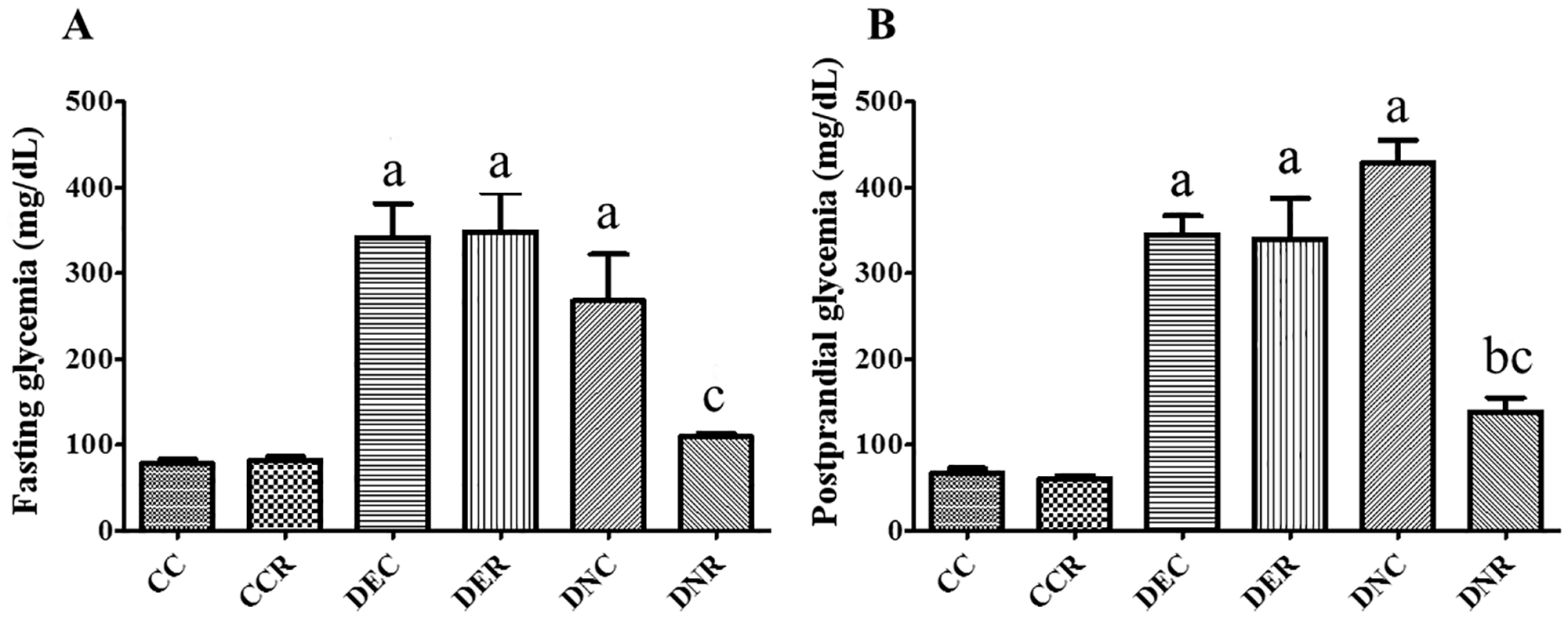
Food restriction glycemia. The glycemia of rats was evaluated in the fasting (A) and postprandial (B) states after 4 months of treatment. Groups: control (CC), control with food restriction (CCR), diabetic+streptozotocin+cafeteria-style diet (DEC); diabetic+streptozotocin+cafeteria-style diet with food restriction (DER), diabetic+streptozotocin+nicotinamide (DNC) and diabetic+streptozotocin+nicotinamide with food restriction (DNR). Results expressed as mean±SE (n = 5/group). *a p<0.05 *vs* CC; b p<0.05 *vs* DEC; c p<0.05 *vs* DNC. One-way ANOVA and Tukey’s *post-hoc* test analysis.

**Fig 4:**
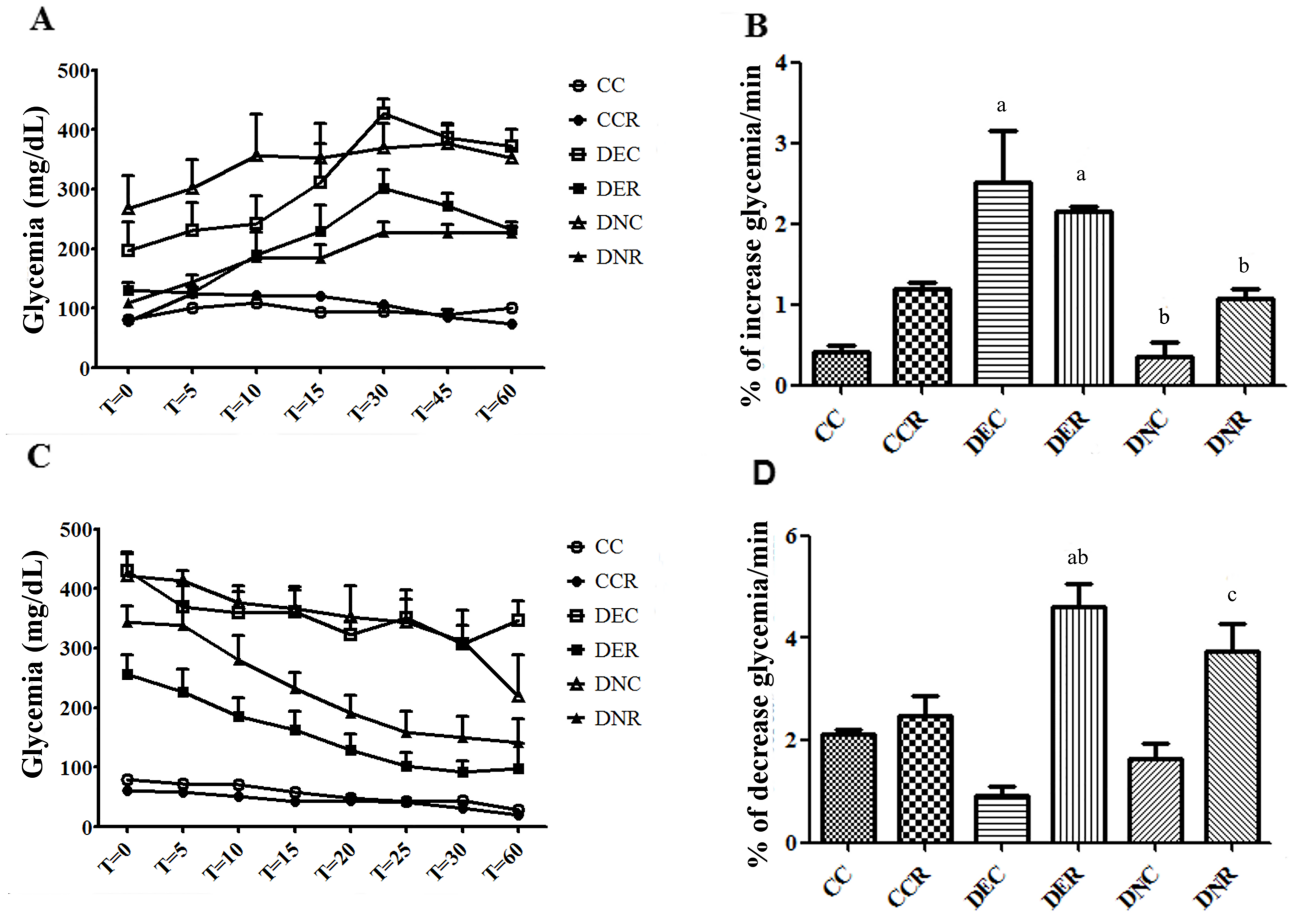
Tolerance to glucose and insulin resistance of rats after food restriction (after 4 months of treatment). (A) Glucose tolerance test (GTT); (B) Rate of increase of glycemia per minute during GTT; (C) Insulin tolerance test (ITT); (D) Rate of blood glucose reduction per minute during ITT. Groups: control (CC), control with food restriction (CCR), diabetic+streptozotocin+cafeteria-style diet (DEC); diabetic+streptozotocin+cafeteria-style diet with food restriction (DER), diabetic+streptozotocin+nicotinamide (DNC) and diabetic+streptozotocin+nicotinamide with food restriction (DNR). Results expressed as mean±SE (n = 5/group). *a p<0.05 *vs* CC; b p<0.05 *vs* DEC; c p<0.05 *vs* DNC. One-way ANOVA and Tukey’s *post-hoc* test analysis.

**Fig 5:**
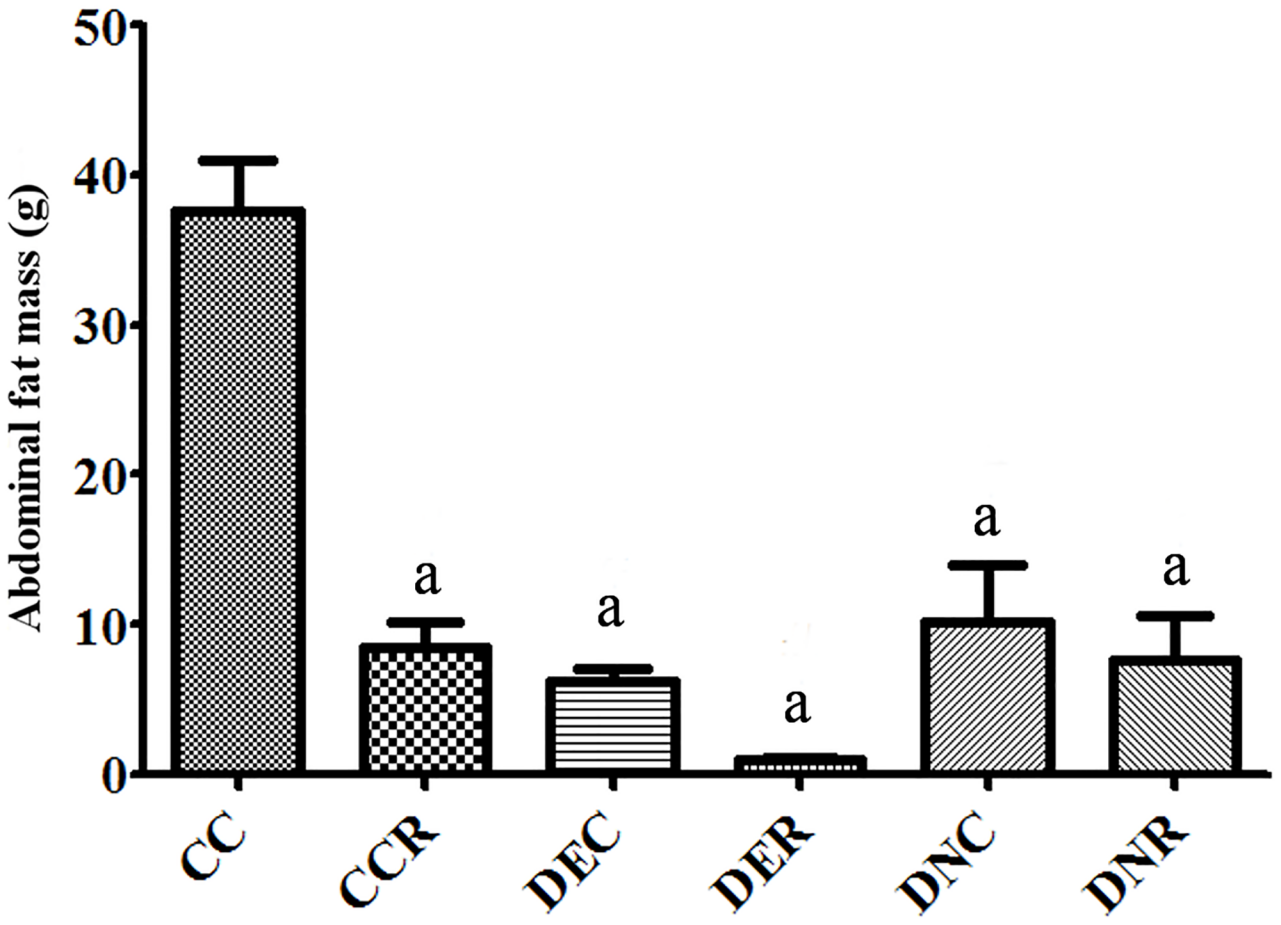
Abdominal fat sum. Added **w**eight of retroperitoneal, mesenteric, periepididimal and subcutaneous fats of rats at the end of the 4 months of treatment for abdominal fat assessment. Groups: control (CC), control with food restriction (CCR), diabetic+streptozotocin+cafeteria-style diet (DEC); diabetic+streptozotocin+cafeteria-style diet with food restriction (DER), diabetic+streptozotocin+nicotinamide (DNC) and diabetic+streptozotocin+nicotinamide with food restriction (DNR). Results expressed as mean±SE (n = 5/group). *a p<0.05 *vs* CC. One-way ANOVA and Tukey’s *post-hoc* test analysis.

The physiological parameters analyzed during treatment are presented in Tables 2 and 3, and Figs 1–5, for each period (pre- and food restriction/FR).

**Table 2:** Average body mass, feed intake and water intake at the end of the first 2 months of treatment (pre-food restriction) of rats. Groups: control (C), diabetic+streptozotocin+cafeteria-style diet (DE) and diabetic+streptozotocin+nicotinamide (DN) rats.

	C	DE	DN
**Body mass (g)**	476.8±12.97	318.3±12.43 ^a^	347.8±10.18 ^a^
**Feed intake (g/day)**	31.9±1.46	48.5±1.58 ^a^	40.5±1.97 ^a^^b^
**Water intake (mL/day)**	69.0±4.64	348.0±13.73 ^a^	156.0±11.49 ^a^^b^

Results expressed as mean±SE (n = 10/group).

^a^ p<0.05 *vs* C;

^b^ p<0.05 *vs* DE.

One-way ANOVA and Tukey’s *post-hoc* test analysis.

**Table 3:** Body mass, feed intake and water consumption of rats after 4 months of treatment (food restriction). Groups: control (CC), control with food restriction (CCR), diabetic+streptozotocin+cafeteria-style diet (DEC); diabetic+streptozotocin+cafeteria-style diet with food restriction (DER), diabetic+streptozotocin+nicotinamide (DNC) and diabetic+streptozotocin+nicotinamide with food restriction (DNR).

	CC	CCR	DEC	DER	DNC	DNR
**Body mass (g)**	489.8±9.31	372.0±9.70	334.8±10.68 ^a^	200.4±8.11 ^a^^b^	351.6±14.18 ^a^	296.8±12.68 ^a^^c^
**Feed intake (g/day)**	27.6±1.63	16.0±0 ^a^	46.8±2.08 ^a^	16.0±0 ^a^^b^	43.4±2.44 ^a^	16.0±0 ^a^^b^^c^
**Water intake (mL/day)**	41.0±5.56	34.0±7.48	152.0±3.74 ^a^	52.0±4.89 ^b^	172.0±16.55 ^a^	48.0±2.00 ^b^^c^

Results expressed as mean±SE (n = 5/group).

^a^ p<0.05 *vs* CC;

^b^ p<0.05 *vs* DEC;

^c^ p<0.05 *vs* DNC.

One-way ANOVA and Tukey’s *post-hoc* test analysis.

#### Pre-food restriction period

After the initial 2 months of treatment, both diabetic groups (DE and DN) showed typical signs of T2DM [10,12,14], such as hyperglycemia (Fig 1), hyperphagia and polydipsia (Table 2), which were significantly higher (p<0.05) in comparison to the values of the control group (C).

The glycemia in the fasted state did not differ between the diabetic groups (Fig 1A). The pre-FR postprandial glycemia (Fig 1B) of the DN group was significantly lower (p <0.05) than the pre-food restriction glycemia of the DE group, but remained higher in relation to group C.

The groups DE and DN did not differ among themselves (p>0.05) in relation to the body mass. On the other hand, higher food intake and water consumption were observed in the DE group compared to DN. In addition to normal water consumption, the DE group presented a mean intake of sugary water (sucrose 32%) of 38.5±5.32 mL, which aided the development of T2DM. Constant diarrhea was also observed only in the animals of the DE group, demonstrating a more severe diabetic state compared to the DN group.

Fig 2A and 2C presents the data obtained from GTT and ITT techniques after the pre-food restriction period. The kGTT (Fig 2B), obtained from GTT, shows that the DE group did not present a higher glycemic elevation rate than the control group (p>0.05). However, the DN group presented a lower rate of glycemic elevation (p<0.05), compared to the other groups. kITT (Fig 2D) on the other hand, demonstrates that both diabetic groups obtained a lower rate of blood glucose reduction (p<0.05) after insulin application, indicating insulin resistance in diabetic animals.

#### Food restriction period

At the end of the 4 months of treatment, the animals of the diabetic groups fed *ad libitum* (DEC and DNC) did not present notable differences in the physiological parameters analyzed, compared to the pre-FR period. Body mass (Table 3) and hyperglycemia (Fig 3) were maintained.

On the other hand, the DEC group had a reduction in the amount of water ingested in this period, thanks to a change from the cafeteria style diet to the standard diet for rodents. Although the total amount of food ingested was similar during both periods, the type of diet offered was determinant in water intake (Table 3). There was maintenance of the physiological parameters of the DNC group, in relation to the pre-food restriction period.

FR, which occurred in months 3 and 4 for the CCR, DER and DNR groups, promoted strong changes (p<0.05) in the physiological parameters. In the CCR group there was a reduction (p<0.05) in body mass only. The FR in diabetic animals previously submitted to streptozotocin and cafeteria-style diet (DER group) promoted a marked reduction of body mass and water intake when compared to DE (before FR) and DEC (diabetic without FR) groups (Table 3). FR also reduced body mass and water intake in DER group. The glycemia of the DER group presented great fluctuations throughout the treatment (S1 Dataset), with glycemic levels sometimes lower when compared to the DE and DEC groups. However, there was no effective reduction of glycemia as a function of FR for the DER group. In relation to the DNR group, FR promoted a more stable reduction (p<0.05) of blood glucose, besides reduced water intake and body mass. Therefore, FR generated a positive effect on glycemia, despite glycemic control being still deficient.

Fig 4A and 4C present the data obtained from GTT and ITT techniques after the food restriction period. FR kGTT (Fig 4B) showed that glucose tolerance group was compromised (p<0.05) only in the DEC relative to the control. The DNC group did not show a change in the rate of increase of glycemia due to a low change in glycemic index, which was already high at the beginning of the test (Fig 4A). kITT (Fig 4D) demonstrated that the DEC and DNC groups showed insulin resistance, although the values were not significantly different (p>0.05) from the CC group. This is due to the low glycemic variation during the test for these groups, indicating reduced insulin action (Fig 4C). In diabetic groups submitted to FR (DER and DNR), glucose tolerance did not show significant improvement in relation to their respective controls (DEC and DNC). On the other hand, insulin sensitivity was improved (p<0.05) in these groups after 2 months of restriction (Fig 4D).

Abdominal fat weighing (Fig 5) showed that both diabetic models (DEC and DNC) and FR promoted a marked reduction (p<0.05) in the abdominal fat mass.

### Evaluation of pancreatic insulin-producing cells

**Fig 6:**
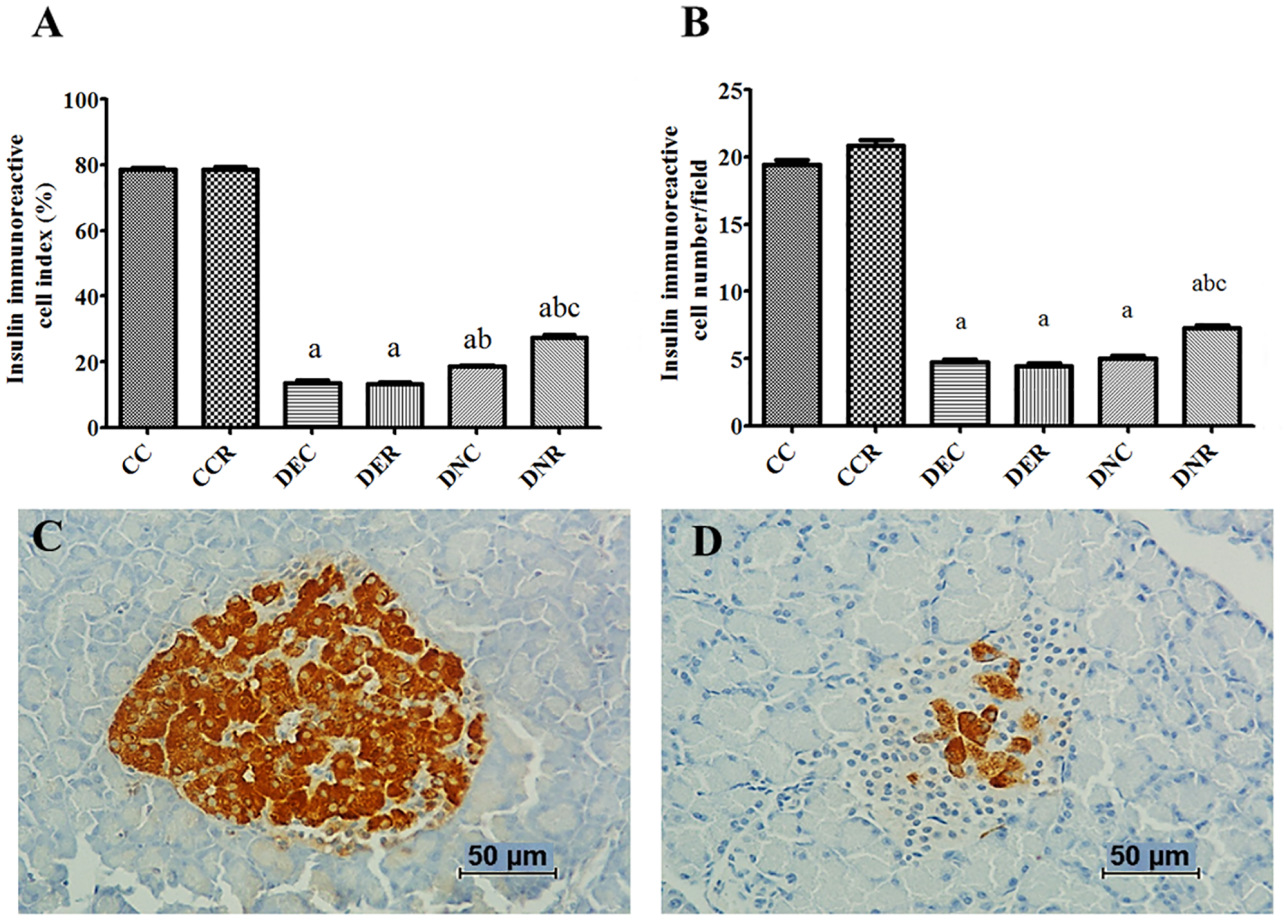
Immunostaining for pancreatic insulin-producing cells of rats after 4 months of treatment. (A) Insulin-producing cells index; (B) Total number of insulin-producing cells; (C) Detail of a pancreatic islet, typical of a non-diabetic animal, immunostained for insulin (200x magnification); (D) Detail of a pancreatic islet, typical of a diabetic animal, immunolabelled for insulin (200x magnification). Groups: control (CC), control with food restriction (CCR), diabetic+streptozotocin+cafeteria-style diet (DEC); diabetic+streptozotocin+cafeteria-style diet with food restriction (DER), diabetic+streptozotocin+nicotinamide (DNC) and diabetic+streptozotocin+nicotinamide with food restriction (DNR). Results expressed as mean±SE (n = 5/group). *a p<0.05 *vs* CC; b p<0.05 *vs* DEC; c p<0.05 *vs* DNC. One-way ANOVA and Tukey’s *post-hoc* test analysis.

The detection of insulin-producing pancreatic islet cells (Fig 6) shows that at the end of the 4 months of treatment, both diabetic groups had reduced (p<0.05) number and proportion of insulin-producing cells in the pancreatic islet. FR was not able to improve this parameter.

### Evaluation of blood insulin

**Fig 7:**
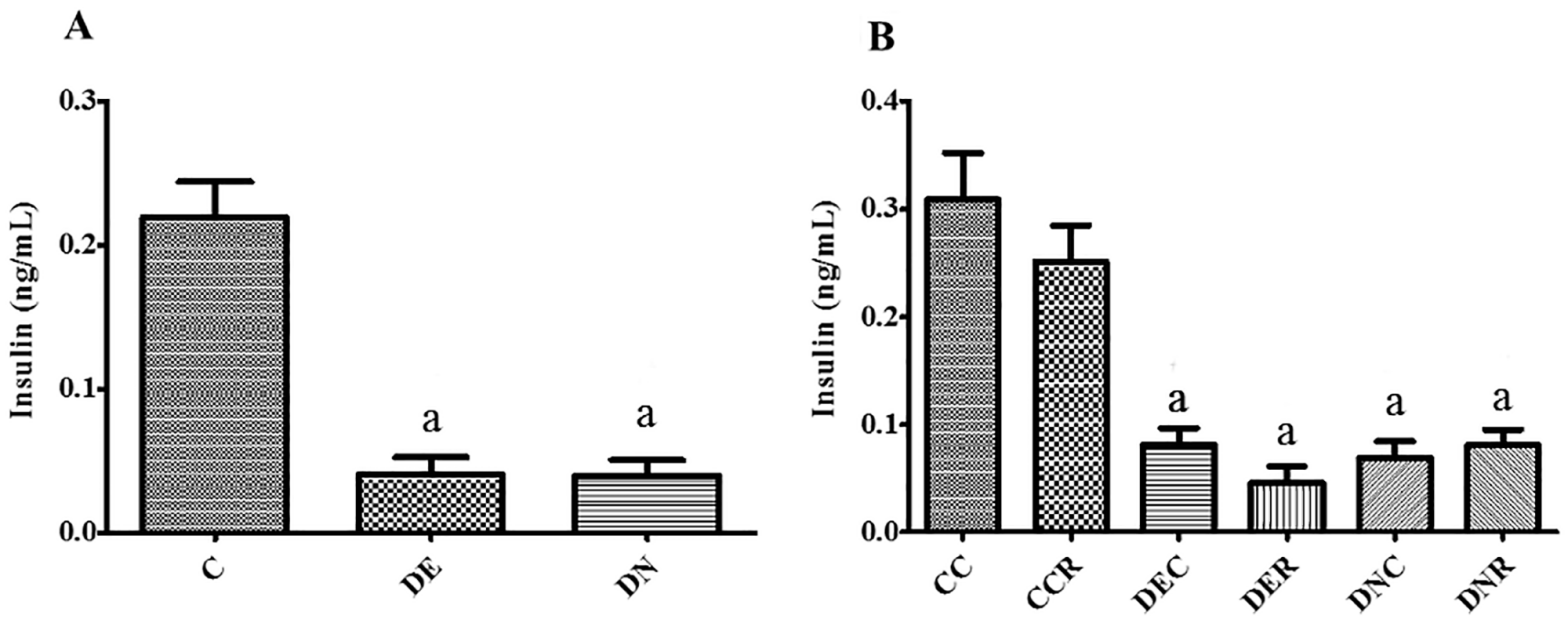
Radioimmunoassay for blood insulin. (A) Amount of immunolabelled insulin in blood of rats after 2 months of treatment for groups control (C), diabetic+streptozotocin+cafeteria-style diet (DE) and diabetic+streptozotocin+nicotinamide (DN). (B) Amount of immunolabelled insulin in blood of rats after 4 months of treatment for groups control (CC), control with food restriction (CCR), diabetic+streptozotocin+cafeteria-style diet (DEC); diabetic+streptozotocin+cafeteria-style diet with food restriction (DER), diabetic+streptozotocin+nicotinamide (DNC) and diabetic+streptozotocin+nicotinamide with food restriction (DNR). Results expressed as mean±SE (n = 3-5/group). *a p<0.05 *vs* CC; b p<0.05 *vs* DEC; c p<0.05 *vs* DNC. One-way ANOVA and Tukey’s *post-hoc* test analysis.

Radioimmunoassay performed in the serum after the initial 2 months of treatment (pre-FR—Fig 7A) shows that the insulin present in the blood was reduced (p<0.05) in all diabetic groups, in relation to the control groups. This characteristic was maintained after 4 months of treatment (Fig 7B), without interference of FR in the animals.

### Biochemical parameters

#### Pre-food restriction period

Table 4 presents the biochemical profile of the blood of the animals after the pre-FR period. Corroborating the diabetic state, there was an increase (p<0.05) in the glycation levels, evaluated through the fructosamine, in both diabetic groups (DE and DN), comparing to group C. Also, there was a rise (p<0.05) of the enzymes AST and ALT, suggesting hepatic damage in both diabetic groups. Elevated levels of alkaline phosphatase also occurred in both T2DM models, although levels of this enzyme were lower (p<0.05) in the DN group than in the DE group. Changes in blood total proteins were only observed in the DN group, which were reduced (p<0.05) when compared to the control group. After 2 months of T2DM, the DE group developed dyslipidemia, with elevated levels of triglycerides and total cholesterol. On the other hand, the DN group did not show changes related to the lipid profile.

**Table 4:** Biochemical parameters from pre-food restriction period (2 months of treatment). Total proteins, fructosamine, triglycerides, cholesterol, AST, ALT and alkaline phosphatase from rats’ blood were evaluated. Groups: control (C), diabetic+streptozotocin+cafeteria style diet (DE), diabetic+streptozotocin+nicotinamide (DN).

	C	DE	DN
**Total proteins (g.dL**^**-1**^**)**	6.95±0.16	6.88±0.15	5.78±0.07 ^a^^b^
**Fructosamine (mg.dL**^**-1**^**)**	0.57±0.04	1.27±0.04 ^a^	1.16±0.04 ^a^
**Triglycerides (mg.dL**^**-1**^**)**	106.7±7.08	285.4±77.30	69.8±9.73 ^b^
**Total cholesterol (mg.dL**^**-1**^**)**	77.6±5.04	117.8±9.16 ^a^	85.2±6.96 ^b^
**AST (U.L**^**-1**^**)**	22.8±0.75	198.1±14.18 ^a^	83.5±9.39 ^a^^b^
**ALT (U.L**^**-1**^**)**	41.40±2.04	309.4±17.30 ^a^	138.8±39.72 ^a^^b^
**Alkaline phosphatase (U.L**^**-1**^**)**	113.6±9.46	639.9±12.47 ^a^	372.6±65.27 ^a^^b^

Results expressed as mean±SE (n = 5/group).

^a^ p<0.05 *vs* C;

^b^ p<0.05 *vs* DE;

One-way ANOVA and Tukey’s *post-hoc* test analysis.

#### Food restriction period

Table 5 shows the blood biochemical profile after 4 months of treatment. The evaluation of fructosamine indicated a maintenance of high glycation levels in the DEC and DNC diabetic groups compared to DE and DN groups, and when compared to CC group (p<0.05). FR was able to reduce fructosamine levels in both diabetic models (DER and DNR).

**Table 5:** Biochemical parameters from food restriction period (after 4 months of treatment). Total proteins, fructosamine, triglycerides, cholesterol, AST, ALT and alkaline phosphatase were evaluated from the blood of rats. Groups: control (CC), control with food restriction (CCR), diabetic+streptozotocin+cafeteria-style diet (DEC); diabetic+streptozotocin+cafeteria-style diet with food restriction (DER), diabetic+streptozotocin+nicotinamide (DNC) and diabetic+streptozotocin+nicotinamide with food restriction (DNR).

	CC	CCR	DEC	DER	DNC	DNR
**Total proteins (g.dL**^**-1**^**)**	5,32±0,19	4,96±0,21	5,5±0,3	5,58±0,59	5,84±0,18	5,85±0,13
**Albumin (g.dL**^**-1**^**)**	2,68±0,05	3,30±0,06 ^a^	3,07±0,07 ^a^	3,16±0,04 ^a^	1,88±0,08 ^a^^b^	2,43±0,02 ^c^
**Fructosamine (mg.dL**^**-1**^**)**	0,96±0,05	0,90±0,05	1,80±0,07 ^a^	1,20±0,14 ^b^	1,68±0,13 ^a^	1,38±0,09 ^c^
**Triglycerides (mg.dL**^**-1**^**)**	137,3±17,55	41,00±2,93 ^a^	126,4±20,10	26,0±3,29 ^a^^b^	68,1±4,91 ^a^^b^	45,38±4,90 ^a^
**Total cholesterol (mg.dL**^**-1**^**)**	58,8±1,73	57,1±4,14	77,0±3,76	44,1±4,10 ^b^	97,5±5,51^a^	84,2±7,69 ^a^
**HDL (mg.dL**^**-1**^**)**	46,5±5,69	40,5±2,81	58,2±4,44	39,0±3,99 ^b^	38,5±1,42 ^b^	31,6±4,67
**VLDL (mg.dL**^**-1**^**)**	27,45±3,50	8,20±0,58 ^a^	25,28±4,02	5,20±0,65 ^a^^b^	13,63±0,98 ^a^^b^	9,07±0,98 ^a^
**AST (U.L**^**-1**^**)**	49,9±1,74	38,2±3,98	110,3±4,64 ^a^	43,0±2,62 ^b^	57,9±2,38 ^b^	37,8±3,93 ^c^
**ALT (U.L**^**-1**^**)**	32,4±3,03	31,4±3,19	178,5±14,55 ^a^	45,8±5,14	75,9±5,18 ^a^^b^	25,8±5,44 ^c^
**Alkaline phosphatase (U.L**^**-1**^**)**	68,63±3,93	143,6±10,15	1064,0±80,58 ^a^	557,9±63,74 ^a^^b^	680,3±52,81 ^a^^b^	256,0±49,84 ^c^

Results expressed as mean±SE (n = 4-5/group).

^a^ p<0.05 *vs* CC;

^b^ p<0.05 *vs* DEC;

^c^ p<0.05 *vs* DNC.

One-way ANOVA and Tukey’s *post-hoc* test analysis.

AST and ALT were only altered in the DEC group, indicating greater tissue damage (p<0.05) in relation to the CC group. There was also a reduction in the levels of these enzymes in the DEC group in relation to the pre-FR period. The return of these enzymes to normal levels, compared to controls, in the DER and DNR groups indicates a positive effect of FR in reducing this type of damage. A similar result was observed in the evaluation of alkaline phosphatase, which was elevated (p<0.05) in the diabetic groups, and decreased (p<0.05) after application of FR in the DER and DNR groups compared to their respective controls.

Total proteins were not altered in the groups after 4 months of treatment. On the other hand, elevation (p<0.05) of albumin levels in the CCR, DEC and DER groups was observed. This may indicate that the model of T2DM allied to the cafeteria-style diet affects albuminuria, while the model of T2DM without dietary change (DNC group) promotes a reduction (p<0.05) of this parameter. These results also indicate that FR may be linked to elevated blood albumin.

The evaluation of the lipid profile indicates that the triglycerides presented reduction (p<0,05) only in the DNC group and in the groups submitted to FR (CCR, DER and DNR). On the other hand, total cholesterol was high (p<0.05) in the DEC, DNC and DNR groups. The DER group presented a significant reduction (p<0.05) when compared to the CC and DEC groups. Regarding cholesterol fractions, it was observed that there were no significant changes in HDL cholesterol levels, but VLDL cholesterol levels were reduced (p<0.05) for all groups, except the DEC group, in relation to the group CC. These data indicate alteration of lipid metabolism associated with T2DM and FR.

### Oxidative status in the plasma

**Fig 8:**
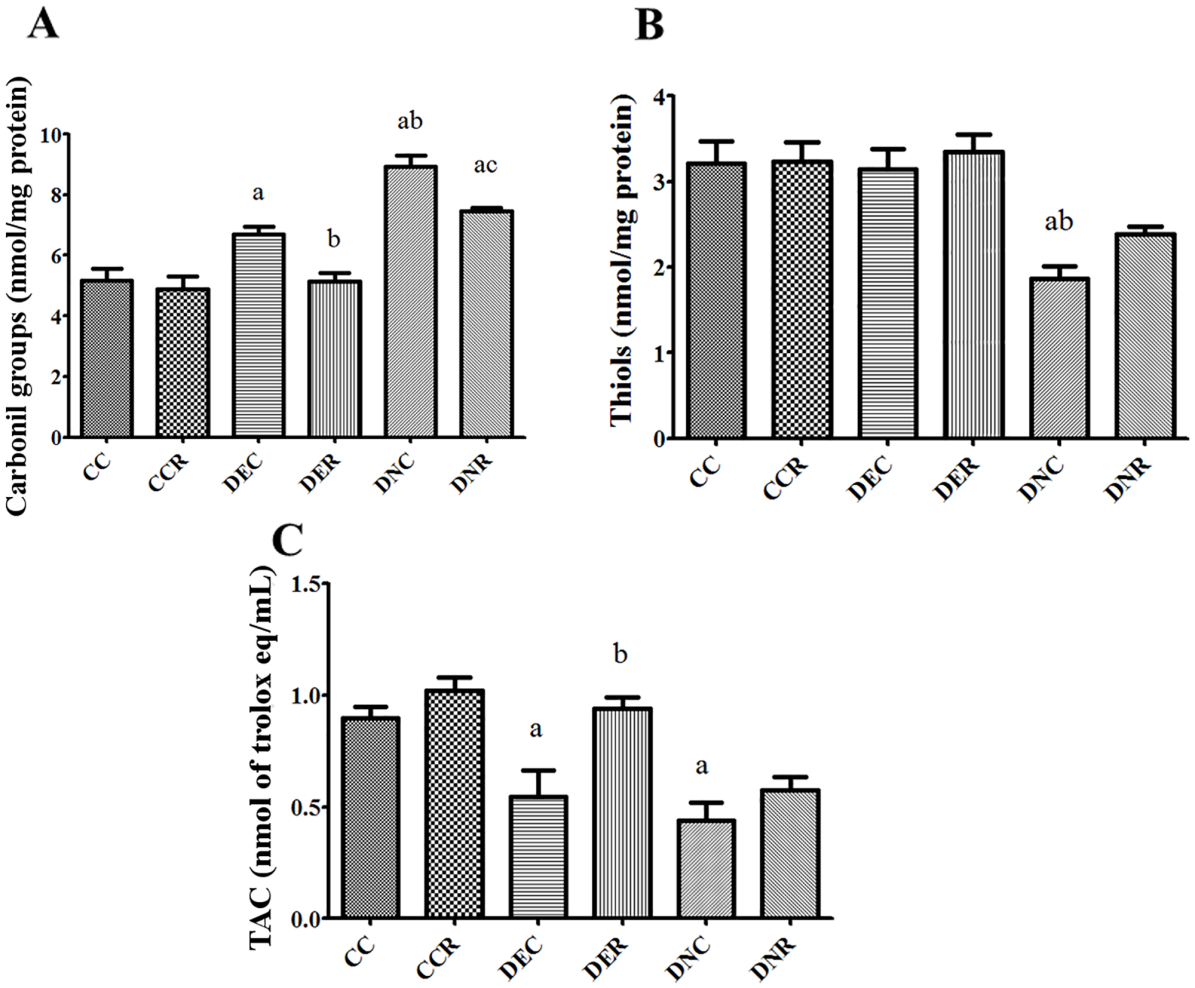
Oxidative stress. Effects of food restriction on the plasmatic oxidative status of type 2 diabetic rats. (A) Protein carbonyl groups, (B) Thiol groups and (C) Total antioxidant capacity (TAC). Oxidative status was assessed at the end of the food restriction period as described in Methods. CC, controls; CCR, controls under food restriction; DEC, diabetic (cafeteria-style diet) rats; DER, diabetic (cafeteria-style diet) rats under food restriction; DNC, diabetic (nicotinamide) rats; DNR, diabetic (nicotinamide) rats under food restriction. Results expressed as mean±SE (n = 4-5/group). *a p<0.05 *vs* CC; b p<0.05 *vs* DEC; c p<0.05 *vs* DNC. One-way ANOVA and Tukey’s *post-hoc* test analysis.

The plasmatic oxidative status was evaluated in the end of the food restriction period. The carbonylation of amino acids has been reported to be the most common oxidative modification in the plasma and thiol protein groups to be the main antioxidant component of the plasma [32]. Thus, the levels of protein carbonyl groups and thiols groups were measured in the plasma of control and diabetic rats. Total antioxidant capacity was additionally and assessed in the plasma of rats. The results are shown in Fig 8. The levels of protein carbonyl groups were 30 and 72% higher in the DEC and DNC diabetic groups, respectively (compared to the control values). Food restriction completely reestablished the levels of protein carbonyl groups in the DER diabetic group, but only partially in the DNR group (compared to the control values). However, it is important to highlight that the levels of protein carbonyl groups in the plasma of the DNC diabetic group were much higher when compared to levels of the DEC diabetic group. Food restriction did not modify the levels of protein carbonyl groups in the plasma of non-diabetic control rats.

The plasmatic levels of thiol groups were reduced only in the DNC diabetic group (-42%; p <0.05), in the same way as the reduction of plasmatic levels of albumin (-31%). Food restriction did not increased the levels of thiol groups in the DNR diabetic group, but it increased the levels of albumin. The total antioxidant capacity (TAC) was strongly reduced in the DEC (-40%) and DNC (-51%) diabetic groups (compared to the controls). Food restriction completely reestablished the antioxidant capacity of the plasma in the DER diabetic group, but it did not improve this parameter in the DNR group.

## Discussion

Weir and Bonner-Weir [35], postulated that human T2DM can be divided into 5 stages, according to the degree of development of the disease and its symptoms, mainly related to changes in pancreatic beta cells. Phase 1 is a normoglycemic period of compensation, with onset of insulin resistance (IR). Phase 5 would characterize the time at which the individual has complete failure of pancreatic beta cells, leading to type 1 diabetes, with total absence of insulin and blood ketosis in the individual. The characteristics of the diabetic models evaluated in our study, such as hyperglycemia, reduced number of pancreatic insulin-producing cells, and presence of insulin resistance (IR) lead us to consider both models as representatives of stage 4, with high glycemic decompensation, IR and loss of function pancreatic beta cells.

The characteristics related to advanced T2DM may generate interest in these models for the study of complications associated with T2DM, such as angiopathies and neuropathies, and allow the use of alternative treatments aimed at improving the diabetic condition.

The diabetic model that uses the streptozotocin (STZ) injection associated with the cafeteria-style diet (adapted from Sahin et al.[15] and Trammel et al.[29]) efficiently simulated the advanced T2DM condition. The use of STZ to induce T2DM is important to replicate some T2DM late metabolic disorders, like hyperglycemia, avoiding the prediabetes situations [7,15,16]. The STZ use assisted in obtaining the advanced T2DM condition and fulfill the purpose of this study, since in literature there are a lot of studies with T2DM in initial stages. The cafeteria-style diet simulated the modern pattern of food intake, with excessive carbohydrates and lower amounts of proteins and fibers. The large amount of carbohydrate associated to this diet was a determinant of the greater proximity of this model to the disease in humans, due to a greater alteration of the lipid profile (triglycerides and cholesterols) and liver damage (AST, ALT and alkaline phosphatase) detected in comparison to the other T2DM model. Characteristics associated with angiopathies (retinopathies) and neuropathies (altered gastrointestinal activity observed as frequent diarrhea, probably derived from oxidative stress) were also present in this model. Models similar to this are found in the literature. Okoduwa et al. [17] found similar characteristics to our model in rats fed with diet added of margarine and sucrose solution. Similar results were found in another study involving induction with hyperlipidic diet, STZ and nicotinamide [10]. Trammel et al. [29] demonstrated that the association of the hyperlipidic diet and STZ was more effective in promoting effects associated with T2DM in mice compared to the isolated hyperlipidic diet, presenting similar characteristics to our model. IR is one of the pathophysiological bases of T2DM [36], and the cafeteria-style diet has already shown potential to generate this resistance [15,16]. The presence of insulin resistance, along with the other characteristics presented, shows that the model that associates STZ and cafeteria-style diet is an alternative for easy simulation of human T2DM in advanced stages, with potential for further development and other studies. This model could also be an alternative to using the conventional cafeteria diet.

Interestingly, Zhou et al. [37] obtained expressive results in certain parameters related to T2DM only with a hypercaloric diet supplied to Sprague-Dawley rats, whereas previous studies demonstrated that only the hyperlipidic diet [14] or cafeteria-style diets alone [15,16,38] were not able to generate T2DM models, despite the presence of obesity or even of prediabetic states. This leads us to conclude that, in general, studies involving diets only in rats are relevant only for the study of prediabetic periods and their prevention. The study for the reversal or cure of T2DM, however, requires more robust and advanced models of T2DM.

An additional feature of our work was the exchange of diets offered to animals, cafeteria style for standard diet only, during the food restriction period (DEC group). The purpose of this amendment was to analyze the effects of altering a hypercaloric diet to a balanced diet over T2DM. The amount of feed ingested was similar for these animals during treatment, but the nutritional quality of the feed was improved. Changes in diet led to a significant reduction in water intake, improvement in overall lipid profile, and in tissue damage markers (AST and ALT). This indicates that only dietary change is sufficient to alleviate such changes, although these levels are still altered in relation to the control. Nevertheless, this isolated alteration was not able to improve the general characteristics of the induced T2DM, such as glycemia, IR and glucose tolerance. Positive results related to dietary changes and/or habits are associated with the improvement of symptoms associated with T2DM, despite the great difficulty of being applied in humans [39]. Female rats showed improvement in several parameters associated with obesity and IR, only with the change from a cafeteria diet to a standard diet for rats [40], reinforcing the benefits of improving eating habits against the development of T2DM.

The strictly chemical induction diabetic model (DN—adapted from Sharma et al.[19]), without dietary intervention, was also efficient in promoting an advanced T2DM. The two consecutive injections of STZ and NIC were applied to ensure the stable T2DM hyperglycemia, since NIC promotes some protection from STZ damage over pancreatic beta cells [12,19]. However, several of the previously mentioned human-associated characteristics were more discreet or distinct when compared to the diet-associated model (DE). The low glycemic variation observed during GTT for this group reflects the lower glycemic fluctuation observed during the whole treatment, allied to high basal glycemia, even after fasting. The data of this model referring to the physiological parameters, insulin, glycation state and oxidative stress are in agreement with the results of the work of Badole et al. [12], with the same model of T2DM, but in Sprague-Dawley rats. On the other hand, these same authors observed a general elevation of the lipid profile of diabetic animals, while our data demonstrated a general reduction for this model. This difference can be due to the difference in lineage or age of the animals.

Therefore, although both models of T2DM have already been used in the literature, the model with dietary intervention is more reliable for the study and extrapolation for the human condition.

A negative point of both models was the absence of obesity, typically associated with T2DM. Although lipid profile alterations were detected, insulin deficiency confirmed by RIA and immunohistochemical techniques seems to have been a limiting factor in the body mass gain in the organism of our diabetic animals. This could also be observed by the reductions of abdominal lipid deposits. The amount of lipid deposits is directly associated with reduction of body mass, which is impacted by the metabolic dysfunction generated by T2DM and/or reduced energy intake.

Lim et al. [21] surprisingly showed that only dietary energy restriction was able to reverse the abnormalities of T2DM in humans, mainly related to pancreatic beta cell function and hepatic insulin sensitivity. However, these benefits were observed only in short-term T2DM, justifying the necessity for studies that simulate long-term T2DM models. Jazet et al. [41] demonstrated that the benefits of the restriction are persistent in the long run. Caloric restriction would also be the main responsible for the benefits of bariatric surgeries on obese and diabetic patients [42,43], as well as by preventing the development of T2DM [44]. Beneficial effects of FR have also been demonstrated [45] and its variations [46–48] in diabetic rats. Our study aimed to complement this scenario, with data on the effect of FR on a more advanced T2DM condition.

In the literature, there are variations of FR with the objective of treating T2DM. In addition to restricted types of nutrients, such as carbohydrates [49] and proteins [48], variations of the restriction periods can be found. Barnosky et al. [50] suggest intermittent fasting and alternate-day fasting as alternatives to common FR. However, since FR would have the same efficacy as intermittent fasting [51], and a smaller number of large daily meals would generate more benefits over T2DM [52], we opted for the choice of continuous FR.

In this study, FR showed potential to improve the diabetic condition. Although the impact on body mass was great, the animals maintained a healthy condition throughout the period. This is supported by total protein levels that were stable throughout the treatment, indicating absence of protein malnutrition. The positive effects of FR on insulin resistance, glycosylation, lipid profile, tissue damage and oxidative stress in diabetic groups are directly related to glucose levels. In the model of T2DM without diet, greater benefit of FR over final glycemia and insulin resistance was observed. The hypoglycemic benefits of FR are mainly related to the negative energy balance that reduces the hepatic glucose production [21]. On the other hand, both models showed elevated oscillation of glycemia during the treatments. This effect may be due to the lower severity of chemically induced T2DM. On the other hand, in relation to biochemical parameters and oxidative stress the FR promoted more significant benefits in T2DM plus cafeteria-style diet.

There are many ways to assess oxidative stress. Analyzing the blood can be an alternative since some oxidation-modified serum components are relatively stable and can be related to the severity of the disease [32]. In the present study, both DEC and DNC diabetic groups presented higher levels of protein carbonyls associated to lower antioxidant capacity of the plasma (Fig 8). These results were already reported in the plasma of poor glycemic control diabetic patients and it has been associated with the development of diabetic complications [53,54]. In fact, the hyperglycemia induces the generation of reactive oxygen species (ROS) in the cells and plasma and proteins are major targets of ROS in the plasma [55]. Thus, the increased levels of protein carbonyl groups in the plasma of both DEC and DNC diabetic animals indicate an increased ROS-mediated injury to serum proteins. The oxidation of plasma thiol groups is itself a manifestation of protein oxidation because the serum albumin contributes with 80% to the plasma thiol groups [56]. Therefore, the lower levels of plasma thiol groups in the DNC diabetic animals possibly reflect an excess of ROS allowing the albumin—SH groups (thiol) to be oxidized [53]. Indeed, the plasmatic thiols serve an antioxidant function and an inverse relation of the plasma thiols and antioxidant capacity of the plasma in diabetic patients is a direct evidence of increased protein oxidation [53]. The plasmatic thiols were not reduced in the DEC diabetic groups, but the protein carbonyl groups increased only slightly (compared to DNC diabetic group). The latter shows the oxidative stress was lesser pronounced in the plasma of DEC diabetic group, in which the reduced antioxidant capacity seems to be not associated to reduced thiols in the plasma. In this regard, plasma contains many antioxidant compounds and the combined action of all these molecules in the plasma represents the antioxidant capacity of the plasma [53].

One of the causes of the oxidative stress observed in this study may be the high glycemic variation observed in our data. It is known that high blood glucose fluctuations, common in patients with uncontrolled T2DM, lead to higher production of ROS and proinflammatory molecules than a regularly elevated glycemia, increasing their circulating levels [57].

Food restriction has been reported to improve the oxidative stress in the plasma of aging rats and in type 2 diabetic rats [47,58–60]. In the present study, food restriction reduced the levels of protein carbonyl groups in the plasma of both DER and DNR diabetic animals and it shows that the ROS-oxidative injury to proteins was reduced in the plasma. The fasting and postprandial glycemia were not improved in DER diabetic group, however, both parameters represent only one punctual dosage in the end of the food restriction period. On the other hand, the levels of fructosamine in the plasma were reduced by food restriction in both DER and DNR diabetic groups, an indicative that the chronic hyperglycemia-induced stress may have reduced in both models of type II diabetes. Regarding the antioxidant status, the total antioxidant capacity (TAC) was improved only in DER diabetic group, in which the levels of albumin and thiols were not diminished in the plasma. The reduced levels of albumin and thiol groups in DNC diabetic rats may occur due to the onset of renal injury, which is associated with an increase in permeability of plasma albumin through a damaged glomerular filtration barrier [61]. The food restriction improved the levels of albumin in the plasma of these animals (DNR), however, it did not improve the thiols and TAC. Therefore, increasing the albumin levels was not enough to increase the antioxidant capacity and it is possible that the sulfhydryl groups of the plasma albumin remained largely in the oxidized state. The latter was also verified in a previous study evaluating the effects of natural oils on the plasmatic oxidative status of rats with adjuvant-induced arthritis and TNBS-induced colitis [62,63].

There is a direct relationship between hyperglycemia and decreased pancreatic islet function, which is reversible [4]. Isolated FR may not be as effective on symptoms related to glycemia and insulin sensitivity, as these benefits are also related to the secretion of incretins produced in the intestine [64]. Troy et al. [65] have demonstrated that bariatric surgery of enterogastric anastomosis type is more efficient in the control of T2DM than simple gastric contrition, a type of forced FR, due to the associated intestinal and hepatic effects. Our results indicate that FR would interfere with the secretion of incretins by the small amount of food that reaches the intestine. Therefore, the benefits on the islet, and consequently on glycemia and insulin resistance, would be reduced.

The distinct forms of T2DM induction can explain the difference in the damages generated by each model of T2DM. The DE groups presented higher glycemic variation and lipid dysfunction, as well as signs of neuropathy, probably due to high carbohydrate intake. On the other hand, the animals of the DN group presented higher oxidative damages and alterations that may reflect nephropathy, common in T2DM [48,66]. Therefore, both these models demonstrate specific metabolic changes that must be considered for possible treatments to be tested.

## Conclusions

Both T2DM models produced the general characteristics of the disease, such as hyperglycemia, insulin resistance, glycation, hepatic damage, hyperphagia and polydipsia. However, some metabolic differences should be observed for its use. The model associated with the cafeteria-style diet (DE) generates greater alterations in the lipid profile, and changes related to neuropathies and retinopathies, being also more susceptible to the benefits of FR. On the other hand, the model without dietary intervention (DN) was more vulnerable to oxidative damage and showed signs of nephropathy, with less benefit of FR. The oxidative stress was increased in both models of type 2 diabetes, but it was more pronounced in the DNC diabetic rats. Food restriction reduced the protein carbonyls in the plasma of both DER and DNR diabetic groups, but the antioxidant capacity was improved only in the plasma of DER group. FR has great potential to improve diabetes-related parameters, although it is not able to reverse the conditions of our models in isolation. Therefore, FR should be associated with other treatments.

## Supporting information

S1 Dataset Excel spreadsheets containing, in separate files, the numerical data for Tables 2–5, and Figs 1–8. (RAR)
